# Miller Fisher syndrome: an updated narrative review

**DOI:** 10.3389/fneur.2023.1250774

**Published:** 2023-08-24

**Authors:** Ciro Maria Noioso, Liliana Bevilacqua, Gabriella Maria Acerra, Paola Della Valle, Marina Serio, Claudia Vinciguerra, Giuseppe Piscosquito, Antonella Toriello, Paolo Barone, Aniello Iovino

**Affiliations:** Neurology Unit, University Hospital “San Giovanni di Dio e Ruggi d'Aragona”, University of Salerno, Salerno, Italy

**Keywords:** Miller Fisher, Miller Fisher syndrome, anti-GQ1b antibody, ataxia, ophthalmoparesis

## Abstract

**Introduction:**

Miller Fisher syndrome (MFS) is considered a rare variant of Guillain-Barré syndrome (GBS), a group of acute-onset immune-mediated neuropathies characterized by the classic triad of ataxia, areflexia, and ophthalmoparesis. The present review aimed to provide a detailed and updated profile of all aspects of the syndrome through a collection of published articles on the subject, ranging from the initial description to recent developments related to COVID-19.

**Methods:**

We searched PubMed, Scopus, EMBASE, and Web of Science databases and gray literature, including references from the identified studies, review studies, and conference abstracts on this topic. We used all MeSH terms pertaining to “Miller Fisher syndrome,” “Miller Fisher,” “Fisher syndrome,” and “anti-GQ1b antibody.”

**Results:**

An extensive bibliography was researched and summarized in the review from an initial profile of MFS since its description to the recent accounts of diagnosis in COVID-19 patients. MFS is an immune-mediated disease with onset most frequently following infection. Anti-ganglioside GQ1b antibodies, detected in ~85% of patients, play a role in the pathogenesis of the syndrome. There are usually no abnormalities in MFS through routine neuroimaging. In rare cases, neuroimaging shows nerve root enhancement and signs of the involvement of the central nervous system. The most consistent electrophysiological findings in MFS are reduced sensory nerve action potentials and absent H reflexes. Although MFS is generally self-limited and has excellent prognosis, rare recurrent forms have been documented.

**Conclusion:**

This article gives an updated narrative review of MFS with special emphasis on clinical characteristics, neurophysiology, treatment, and prognosis of MFS patients.

## 1. Introduction

Miller Fisher syndrome (MFS) is considered a rare variant of Guillain-Barré syndrome (GBS), a group of acute-onset immune-mediated neuropathies characterized by the classic triad of ataxia, areflexia, and ophthalmoparesis. The present review aimed to provide a detailed and updated profile of all aspects of the syndrome through a collection of published articles on the subject ranging from the initial description to recent developments related to COVID-19.

## 2. Methods

We searched PubMed, Scopus, EMBASE, and Web of Science databases and gray literature, including references from the identified studies, review studies, and conference abstracts on this topic. We used all MeSH terms pertaining to “Miller Fisher syndrome,” “Miller Fisher,” “Fisher syndrome,” and “anti-GQ1b antibody.”

## 3. Epidemiology

The worldwide annual incidence of GBS is ~1–2/100,000 inhabitants. Of these, MFS represents a small fraction of the total, with the percentage varying according to the area considered. There is a slight male predominance, and MFS can occur in all age groups. The incidence is higher in Asian countries, where it can reach 15–25% of GBS cases: an 11-year retrospective study in Taiwan estimated a relative incidence of ~18%, and other studies found a 9% incidence in Hong Kong and a 7.7% incidence in Thailand (1–3). It is lower in the West, where it accounts for ~1–7% of GBS cases: an Italian study estimated the incidence in Europe to be between 0.04 and 0.18 cases per 1,00,000 inhabitants or ~6.6% of GBS patients; while a 7% incidence was recorded in Spain (4, 5). Such a low incidence justifies the absence of randomized trials on patients. All currently published studies appear to be retrospective, with very limited case studies.

There is no consensus regarding the classification of GBS and its variants. A more inclusive approach, based on clinical features, was proposed by the GBS Classification Group in 2014, placing both GBS and MFS within a continuous spectrum disorder, considering apparent differences in pathogenesis, treatment, and prognosis (6) (Table 1).

**Table 1:** GBS, MFS, and their subtypes.

	**Clinical features**
**GBS**	
Classic GBS	A form with acute flaccid paralysis of all four limbs
Pharyngeal-cervical-brachial weakness	Localized subtypes of GBS
Paraparetic GBS	
Bifacial weakness with paresthesias	
Acute pharyngeal weakness	Incomplete form of pharyngeal-cervical-brachial weakness
**MFS**	
Classic MFS	A form with acute ophthalmoplegia and ataxia
Acute ophthalmoparesis	Incomplete forms of MFS
Acute ataxic neuropathy	
Acute ptosis	
Acute mydriasis	
Bickerstaff brainstem encephalitis	A form with hypersomnolence, ophthalmoplegia, and ataxia
Acute ataxic hypersomnolence	Incomplete form of BBE with hypersomnolence and ataxia, no weakness

## 4. Clinical features

MFS classically presents itself with a symptomatological triad characterized by ophthalmoparesis, ataxia, and osteotendinous areflexia, appearing in ~80% of patients (7, 8). Ophthalmoparesis, usually bilateral, progresses to complete external ophthalmoplegia in 1–2 weeks. Ataxia, often very severe, may cause an inability to walk without support despite normal strength. Areflexia, a less specific element of the triad (absent in 18%), may also be limited to an isolated body area (9). The symptomatological triad is also frequently associated with the presence of extra signs such as ptosis (60%), facial nerve palsy (30–50%), sensory deficits (20–50%), and hyposthenia (20–25%) (10–13) (Table 2).

**Table 2:** Extra signs associated with the symptomatological triad.

Ptosis	60%
Facial nerve palsy	30–50%
Sensory deficits	20–50%
Hyposthenia	20–25%

Interesting evidence emerges from a retrospective study tracing the 10-year experience of a third-level center. It takes into account 19 cases of MFS documented from 1995 to 2005: epidemiologically, there is a clear male prevalence (M>F 84%), early age onset (36 years on average), and autumn–winter peak (73% of cases), which corresponds with the higher incidence of respiratory infections (URIs). This study showed that the average interval between infection and the onset of neurological symptoms is 7 days (ranging from 1 to 30) and that diplopia is the main onset symptom (63%).

The clinical timing for onset varies according to the symptom considered: ophthalmoparesis appears to be the earliest symptom, presenting itself at ~7 days (variability 1–30 days), followed by ataxia, which on average appears at ~10 days (variability 1–30 days), while areflexia, the symptom with the most variable onset of the three, presents itself on average at 14 days (variability 4–45 days) (14).

A longitudinal assessment of the pathology thus becomes essential for diagnosis.

MFS with atypical manifestations has also been described: possible onset with bilateral internal ophthalmoplegia, unilateral external ophthalmoplegia, bilateral abducent nerve palsy, and isolated bilateral ptosis (15–18).

The literature reports cases of generally bilateral optic neuritis, causing visual impairments with no signs of pain in eye movement, color desaturation, or field deficit. The optic fundi are normal. Orbital magnetic resonance imaging or visual evoked potentials (VEPs) revealed bilateral optic neuropathy (19, 20).

## 5. Pathogenesis

The origin of MFS has been shown to be immunologic. The infectious antecedent appears to be present in the majority of patients: previous infection of the upper respiratory apparatus is most frequent (56–76% of patients). Gastrointestinal infection (4%), typical of classical GBS, and isolated fever (2%) are rarer. MFS associated with autoimmune or neoplastic disorders is also possible (1, 13, 21–24). Additional factors that increase the risk of the disease comprise the utilization of specific medications (such as heroin, isotretinoin, and streptokinase), implementation of TNF-alpha antagonist treatment, bone marrow transplantation, and surgical procedures (25–27).

In this light, the higher incidence of MFS in the Far East could also be related to the higher rate of infections. Pathogens most frequently implicated are *Campylobacter jejuni* (21%) and *Haemophilus influenzae* (8%) (22, 28). In most cases, the pathogen responsible is not known (28).

The infectious etiology had already been suspected by Miller Fisher and then corroborated by Koga (8, 28). The underlying mechanism seems to be that of “molecular mimicry:” The immune system's activation of the lipo-oligosaccharides (LOS) present on the membrane of these pathogens, which are similar in shape to gangliosides (GQ1b, GM1, and GD1a), would lead to the production of autoantibodies. If the antibody produced is GM1 or GD1b, the classic form of GBS is produced, whereas if it is GQ1b, MFS is produced (29).

Antibodies against GQ1b have a sensitivity of 85% and a specificity of 100% (30).

**Figure 1:**
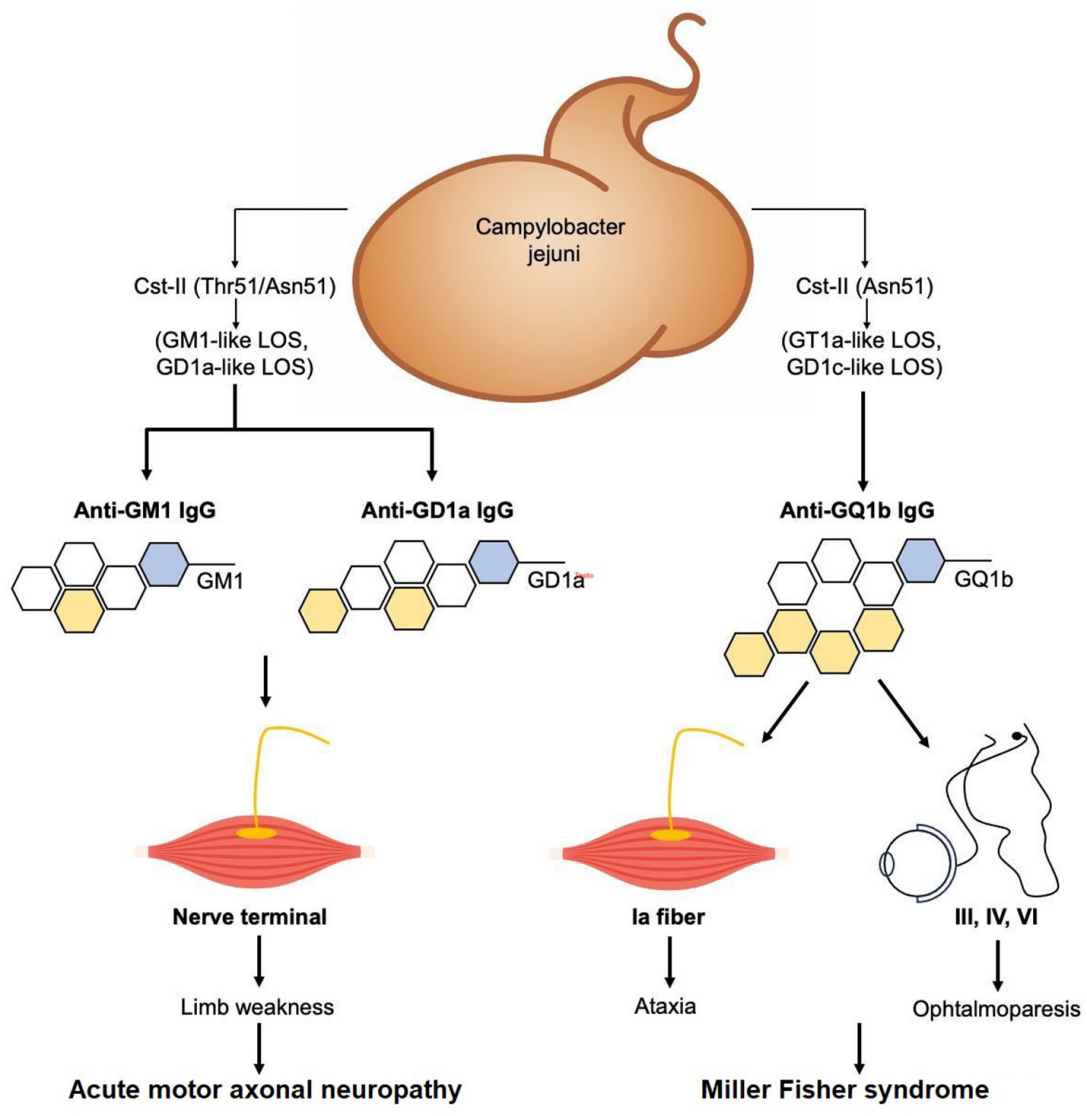
The underlying mechanism of MFS seems to be that of “molecular mimicry.” The immune system's activation of the lipo-oligosaccharides (LOS) present on the membrane of some pathogens, most frequently *Campylobacter jejuni*, which are similar in shape to gangliosides (GQ1b, GM1, and GD1a), would lead to the production of autoantibodies. If the antibody produced is GM1 or GD1b, the classic form of GBS with acute motor axonal neuropathy is produced, whereas if it is GQ1b, MFS is produced (29). GQ1b is a ganglioside present in the paranodal myelin, mainly at the level of oculomotor nerves (III-IV and VI cranial nerves), dorsal root ganglia (DRG), and fibers of neuromuscular spindles. The localization of the ganglioside explains the symptomatological triad of patients.

GQ1b is a ganglioside present in the paranodal myelin, mainly at the level of oculomotor nerves (III-IV and VI cranial nerves), dorsal root ganglia (DRG), and fibers of neuromuscular spindles (Figure 1).

The localization of the ganglioside explains the symptomatological triad of patients with ophthalmoparesis, ataxia, and areflexia due to the involvement of the muscle spindles (1). The possible involvement of the optic nerve with optic neuritis is explained by the presence of the GQ1b epitope in the optic nerve (31).

The passage of the autoantibodies would take place at the level of the blood–nerve barrier of the neuromuscular junctions (NMJs), an area rich in gangliosides, which are dependent on rapid cell turnover for its function, as is also shown by histological analyses (32).

GQ1b is expressed in the NMJs of the oculomotor nerves and of muscle spindles and serves as a target for autoantibodies producing the characteristic outlook of MFS.

Antibodies against GQ1b ganglioside are present in Bickerstaff brainstem encephalitis (BBE) as well as MFS. BBE is an even rarer related condition in which ataxia and ophthalmoparesis are accompanied by impaired consciousness and hyperreflexia. The presence of these symptoms points to anti-GQ1b antibodies having pathologic effects in the central nervous system as well as in the cranial and peripheral nerves.

At the level of the nodes of Ranvier, three distinct areas can be identified:

- Juxtaparanodal region, where the compacted myelin is tightly attached to the axolemma.
- Paranodal region, where the myelin is attached to the axolemma but not organized as a compact structure.
- The node of Ranvier, in which the axolemma is not lined with myelin and is in direct contact with the extracellular fluid, despite being covered by the microvillous Schwann cells.

**Figure 2:**
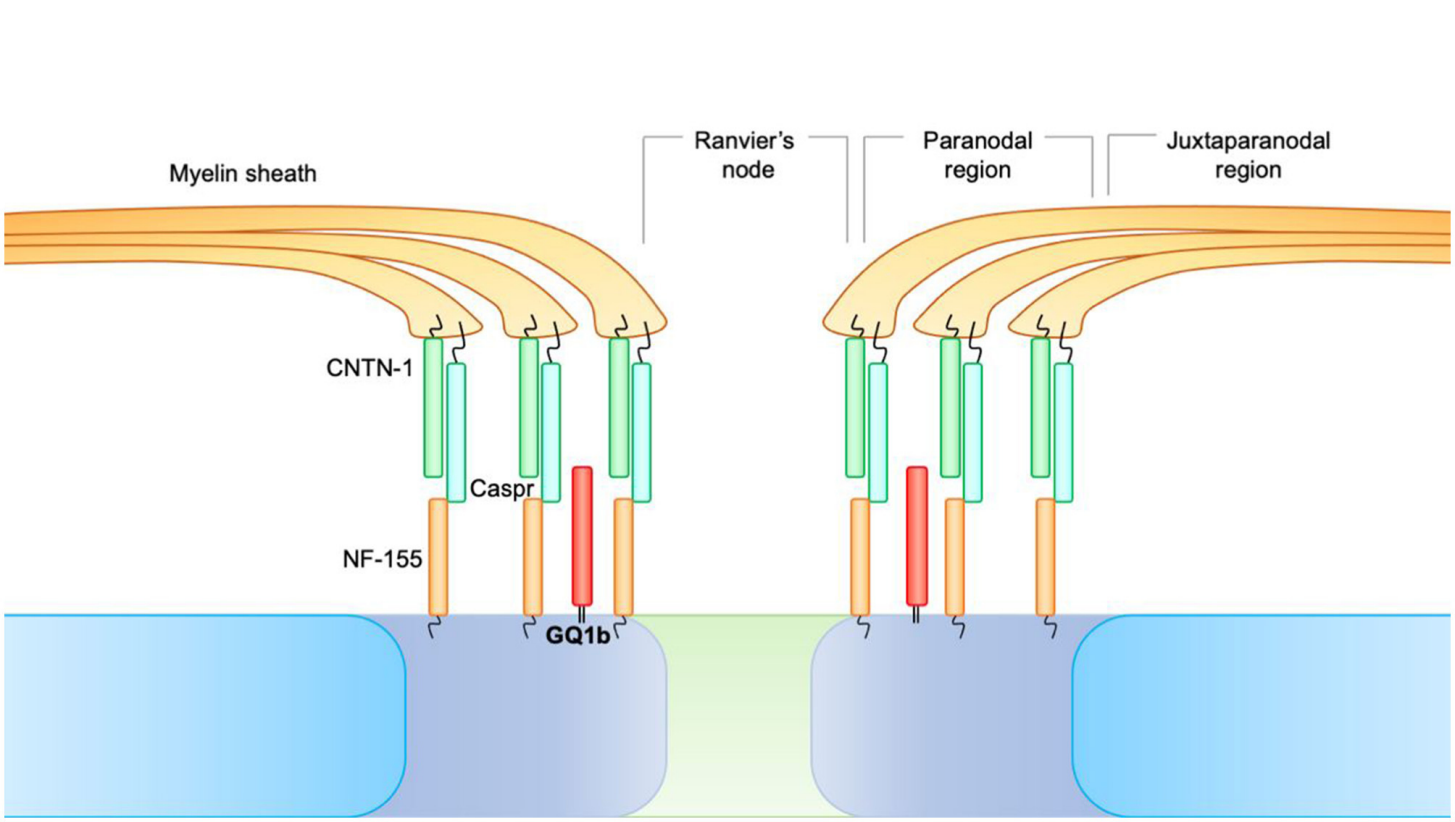
At the level of the nodes of Ranvier, three distinct areas can be identified. 1. Juxtaparanodal region, where the compacted myelin is tightly attached to the axolemma. 2. Paranodal region, where the myelin is attached to the axolemma but not organized as a compact structure. 3. The node of Ranvier, in which the axolemma is not lined with myelin and is in direct contact with the extracellular fluid, despite being covered by the microvillous Schwann cells. At the level of the paranode, there is a formation consisting of Contactin-1 (CNTN1) and CASPR, expressed by neurons, which bind the NF-155 counterpart and are of glial origin. This paranodal axon-glial formation is responsible for ion channel clustering, propagating the action potential, and blocking the lateral diffusion of membrane proteins in myelinated nerve fibers.

Fundamental to longitudinal impulse conduction is the paranodal region. At the level of the paranode, there is, in fact, a formation consisting of Contactin-1 (CNTN1) and CASPR, expressed by neurons, which bind the NF-155 counterpart, and is of glial origin. This paranodal axon-glial formation is responsible for ion channel clustering, propagating the action potential and blocking the lateral diffusion of membrane proteins in myelinated nerve fibers (Figure 2).

GQ1b acts by stabilizing this paranodal formation (33). GQ1b autoantibodies, acting against the paranodal formation, cause acute blockade of anterograde propagation of the electrical impulse, justifying the acute symptomatic onset (29).

Certain cases have been reported of anti-glutamic acid decarboxylase (GAD) seropositive patients, including those with anti-GQ1b seronegative forms (34). Anti-GAD plays an uncertain role in the pathogenesis of MFS. Titers, though inferior to that found in stiff person syndrome, appeared increased in these patients' serum. A progressive decrease in titers is associated with clinical and electrophysiological improvement. The pathogenetic role of these antibodies would involve causing the disruption of GABA synthesis through GAD inhibition in the brainstem and cerebellum (35–38). GAD is the rate-limiting enzyme for the synthesis of the inhibitory gamma-aminobutyric acid (GABA) (38). Supporting evidence of a broader role of anti-GAD antibodies in the pathogenesis of MFS is the case, described by Javaid et al., of a patient presenting seronegative BBE with anti-GAD seropositivity (39).

### 5.1. Relation to SARS-CoV-2

COVID-19 (coronavirus disease 2019) appears to be a possible infective antecedent to MFS.

COVID-19 is associated with several systemic symptoms, the most frequent being gastrointestinal, cardiovascular, dermatological, and neurological symptoms (40).

A retrospective study on 214 hospitalized patients reported neurological complications in 36% of the cases examined with involvement of the CNS (24.8%) greater than the peripheral nervous system (8.9%) (41). These varied from lighter forms such as headache, dizziness, myalgia, and anosmia to more severe symptoms such as encephalopathy, encephalitis, necrotizing hemorrhagic encephalopathy, stroke, epileptic seizures, and Guillain-Barré syndrome (40).

Infection may be either hematogenous or via retrograde neural propagation along the olfactory pathway in which ACE2 (angiotensin-converting enzyme 2) functional receptor is more present (42).

An Italian study demonstrated how the percentage of GBS patients following SARS-CoV2 infection substantially increased during the pandemic compared to the previous 30 years (43–45).

According to the ALBACOVID registry, GBS patients accounted for 0.5% of hospitalizations due to COVID-19 (46). A systematic review by Aladawj found that, out of 99 COVID-19-related GBS cases described in the literature, nine presented the Miller Fisher variant (47).

Although a rare occurrence, multiple cases of MFS following COVID-19 vaccination have been described across several countries (48–50). Unlike GBS associated with vaccination, it does not appear to be related to the viral vector vaccination (51).

## 6. Diagnosis

The diagnosis of MFS is based on clinical aspects and may be supported by laboratory investigations. Various “formes frustes” and overlap syndromes have been reported to date, thereby expanding the understanding of this clinical spectrum. In the cerebrospinal fluid (CSF), hyperproteinorrachia, a marker of GBS, may not be an early finding and may not be present in all cases, i.e., it can be found in ~47% of patients. Of these patients, the incidence of hyperproteinorrachia increases progressively over the first 3 weeks from 66% in week 1 to 82% in week 3 (1).

More frequent is the finding in overlap syndromes: In these syndromes, the incidence of hyperproteinorrachia is similar to that of classic GBS (1).

In contrast, anti-GQ1b antibodies are found in the CSF of almost 85% of patients with MFS (1). This percentage is higher in other studies (22). The anti-GQ1b Ab dosage appears to be much more useful than the proteins in the CSF during the 1st week. Ab anti-GD1a (28%) and Ab anti-GM1 (15%), whose pathogenic role has not yet been demonstrated, may also be found (52).

Anti-GQ1b antibodies are also present in the forms of GBS in overlap with ophthalmoparesis in acute isolated ophthalmoplegia and in BBE: these forms constitute the “Anti-GQ1b spectrum” (53).

Although MFS belongs to the group of peripheral neuropathies, the hypothesis of concomitant central involvement has been put forward by different authors (54–56).

Fisher considered ataxia as the manifestation of an unusual peripheral neuron lesion but also reported it as the cerebellar type as it was disproportionate to sensory loss (8).

Some authors reported a cerebellar type of ataxia and supranuclear ophthalmoplegia with Bell's sign positive in MFS and suggested additional involvement of the central nervous system (57).

As proof of this finding, a study was conducted on MFS patients using 18F-fluorodeoxyglucose-positron emission tomography (FDG-PET) to determine the involvement of the central nervous system. Cerebral glucose metabolism in 10 patients with MFS was compared with that of 60 age- and sex-matched normal controls using PET. Group analyses described increased metabolism in the cerebellar vermis, bilateral cerebellar hemispheres, pontine tegmentum, midbrain tectum, right inferior frontal cortex (Brodmann area 47), and left thalamus and decreased metabolism in the bilateral occipital cortices (Brodmann area 18 and 19). The hypermetabolism in those disorders has been attributed to inflammatory changes, and stereotaxic biopsy indeed revealed inflammation with lymphocytic infiltrations and reactive gliosis in one patient (58).

Hypermetabolism of the right inferior frontal cortex would be associated with hyperactivation to adjust locomotion and limb movements, which are impaired by diplopia and ataxia. A similar finding of sustained prefrontal hyperactivity is also observed during ataxic gait in patients with infratentorial stroke, which also suggests a compensatory mechanism for impaired locomotor control (59).

Hypometabolism of the associative visual cortex (Brodmann area 18 and 19) has been interpreted as an adaptive functional suppression of the cortical responses to deranged visual inputs (60).

MFS is not generally associated with abnormalities in brain imaging. Hyperintensity in T2W at the level of the brainstem and the cranial and spinal nerve roots can be found in 2% of cases, providing evidence supporting central involvement (61–63).

Electrophysiologically, the neurophysiological alterations are generally minor when compared to the ones presented in GBS. The hallmarks typical of acquired demyelinating polyneuropathies such as reduced motor conduction velocity (MCV), marked temporal dispersion, and conduction blocks are not present here. Motor and sensory conduction studies are generally within normal limits. Sensory neuropathy characterized by amplitude reduction disproportionate to the slowing of sensory conduction velocities or prolongation of the distal latencies may appear (64, 65).

More indicative is the study of late responses, which allows the evaluation of the most proximal segment of nerves such as plexuses and roots, inaccessible to routine nerve conduction studies (NCSs). The F response, a late motor response, generally appears to be normal; in rare cases, it is possible to find the prolongation or absence of this reflex (65).

A constant abnormality in patients with MFS appears to be the absence of the H reflex (66, 67). The disappearance of this late reflex, the neurophysiological equivalent of the myotatic reflex, appears to be ascribable to the impairment of the fibers of the neuromuscular spindle that present the GQ1b epitope on the surface, which is responsible for ataxia and areflexia. These fibers cannot be investigated with routine NCSs, but only in mixed nerve studies and with the H reflex. Recovery from this neurophysiological alteration is associated with patient recovery (13).

## 7. Therapy

As MFS is a rare condition, there are no randomized, double-blind, placebo-controlled trials on treatment and retrospective studies are controversial.

A large retrospective study of 92 patients, including 28 treated with intravenous immunoglobulin (IVIG) (0.4 g/kg/day × 5 days), 23 treated with plasma exchange (PLEX; 2–6 cycles, average 4), and 41 untreated, retrospectively assessing the time of recovery from ophthalmoplegia and ataxia, showed that the survival rate was ~100%. The resolution of areflexia could not be retrospectively assessed as it did not alter the patients' day-to-day living. IVIG slightly improved the patient's condition and recovery time by reducing the binding of anti-GQ1b antibodies and limiting the pathologic effects of the antibodies (68). Plasmapheresis would neither improve symptomatology nor recovery time (69).

The natural history of the disease is self-limiting. Therapy would not affect the patient's outcome. The natural course of MFS is characterized by excellent recovery.

Although rare cases of recurrent MFS have also been recorded in the literature, a review by Ishii et al. found that 12% of MFS patients presented a recurrent form (70). Clinical, electrophysiological, and laboratory analyses of these patients presented no apparent differences from that of non-recurrent MFS. Epidemiological analysis revealed a younger median onset age (22 years as opposed to 37 years in non-recurrent forms). Although there is ample variability in the number of recurrences, all cases examined presented similar symptoms to the first occurrence, though less severe (70).

As reported by Chida, the recurrence of MFS in these patients may depend on a gene polymorphism: the presence of HLA-DR2 antigen is significantly higher than that in patients with monophasic MFS and healthy controls (71).

MFS was found to overlap with other disorders within 7 days of onset in ~50% of cases: classic GBS (15%), pharyngeal-cervical-brachial GBS (23%), and BBE (12%), which may place MFS within a continuous spectrum disorder (6, 72, 73). Early recognition and IVIG or PLEX are recommended in these cases (6, 72, 74, 75).

The average resolution time of symptoms varies depending on the symptom considered: recovery from ataxia takes 35 days (10–121), ophthalmoplegia takes 3 months (93 days, 18–244), and areflexia of the 3 symptoms is the most variable (64 days, 10–650). At outpatient follow-up, all patients were free of ataxia, ophthalmoplegia, and areflexia after an average of 6 months, returning to their normal activities with no functional limitations (14).

## 8. Seronegative forms

Particular attention should be paid to the clinical forms of MFS that show CSF negativity for GQ1b.

As shown in a study published in the Journal of Neurology in 2012, 12% of MFS are seronegative and ~70% of these are positive for GM1. These forms, clinically similar to GQ1b+ forms, differ in that they almost exclusively affect men and for a higher frequency of gastroenteritis as an infectious antecedent (76).

These forms are generally paraneoplastic and are more frequently associated with Burkitt lymphoma, diffuse large B cell lymphoma, chronic lymphocytic leukemia, Hodgkin's disease, and leptomeningeal signet ring cell carcinomatosis of lung cancer (21, 77, 78).

## 9. Conclusion

This article aimed to provide an updated narrative review of the clinical features, neurophysiology, treatment, and prognosis of patients with MFS.

MFS is a rare, immune-mediated disorder that presents itself with a classical triad of ataxia, areflexia, and ophthalmoplegia. Apart from these classic clinical signs, other signs may be associated with it, such as optic neuritis, facial palsy, limb weakness, and superficial sensory loss.

Anti-ganglioside GQ1b antibodies play a role in the pathogenesis of the syndrome. These antibodies can be found in ~85% of patients, peaking in the 1st week, whereas albuminocytological dissociation in the cerebrospinal fluid (CSF) appears later. Electrophysiologically reduced sensory nerve action potentials and absent H reflexes are also characteristic. The natural course of MFS is characterized by excellent recovery.

Although no abnormalities are usually found in MFS by routine neuroimaging, contrast enhancement in T2-weighted sequences of nerve roots and signs of the involvement of the central nervous system have been described in some cases, supporting the hypothesis of central and peripheral involvement.

## Author contributions

All authors listed have made a substantial, direct, and intellectual contribution to the work and approved it for publication.
